# Mitochondrial ncRNA targeting induces cell cycle arrest and tumor growth inhibition of MDA-MB-231 breast cancer cells through reduction of key cell cycle progression factors

**DOI:** 10.1038/s41419-019-1649-3

**Published:** 2019-05-29

**Authors:** Christopher Fitzpatrick, Maximiliano F. Bendek, Macarena Briones, Nicole Farfán, Valeria A. Silva, Gino Nardocci, Martín Montecino, Anne Boland, Jean-François Deleuze, Jaime Villegas, Claudio Villota, Verónica Silva, Lorena Lobos-Gonzalez, Vincenzo Borgna, Eric Barrey, Luis O. Burzio, Verónica A. Burzio

**Affiliations:** 1 Fundación Ciencia & Vida/Andes Biotechnologies SpA, 7780272 Santiago, Chile; 2 Department of Biological Sciences, Faculty of Life Sciences, Universidad Andrés Bello, 8370134 Santiago, Chile; 3 Center for Biomedical Research and FONDAP Center for Genome Regulation, Faculty of Life Sciences and Faculty of Medicine, Universidad Andrés Bello, 8370134 Santiago, Chile; 4 Centre National de Recherche en Génomique Humaine (CNRGH), Institut de Biologie François Jacob, CEA, Evry, France; 5 Andes Biotechnologies Global Inc., Burlingame, CA USA; 6 School of Nutrition and Diet, Faculty of Health, Universidad Bernardo O’Higgins, Santiago, Chile; 7 Center for Regenerative Medicine, Faculty of Medicine, Clínica Alemana & Universidad del Desarrollo, Santiago, Chile; 8 Faculty of Medical Sciences, Universidad de Santiago de Chile, Santiago, Chile; 9 INRA, Génétique Animale et Biologie Intégrative UMR1313, AgroParisTech, Université Paris-Saclay, Jouy-en-Josas, France; 10 Present Address: Valparaíso Interdisciplinary Neuroscience Center, Faculty of Sciences, Universidad de Valparaíso, Valparaíso, 2360102 Chile; 11 Present Address: Center for Veterinary Medicine, Faculty of Life Sciences, Universidad Andrés Bello, Santiago, Chile

**Keywords:** Breast cancer, Mitosis, Target validation, Long non-coding RNAs, miRNAs

## Abstract

The family of long noncoding mitochondrial RNAs (ncmtRNAs), comprising sense (SncmtRNA), and antisense (ASncmtRNA-1 and ASncmtRNA-2) members, are differentially expressed according to cell proliferative status; SncmtRNA is expressed in all proliferating cells, while ASncmtRNAs are expressed in normal proliferating cells, but is downregulated in tumor cells. ASncmtRNA knockdown with an antisense oligonucleotide induces massive apoptosis in tumor cell lines, without affecting healthy cells. Apoptotic death is preceded by proliferation blockage, suggesting that these transcripts are involved in cell cycle regulation. Here, we show that ASncmtRNA knockdown induces cell death preceded by proliferative blockage in three different human breast cancer cell lines. This effect is mediated by downregulation of the key cell cycle progression factors cyclin B1, cyclin D1, CDK1, CDK4, and survivin, the latter also constituting an essential inhibitor of apoptosis, underlying additionally the onset of apoptosis. The treatment also induces an increase in the microRNA hsa-miR-4485-3p, whose sequence maps to ASncmtRNA-2 and transfection of MDA-MB-231 cells with a mimic of this miRNA induces cyclin B1 and D1 downregulation. Other miRNAs that are upregulated include nuclear-encoded hsa-miR-5096 and hsa-miR-3609, whose mimics downregulate CDK1. Our results suggest that ASncmtRNA targeting blocks tumor cell proliferation through reduction of essential cell cycle proteins, mediated by mitochondrial and nuclear miRNAs. This work adds to the elucidation of the molecular mechanisms behind cell cycle arrest preceding tumor cell apoptosis induced by ASncmtRNA knockdown. As proof-of-concept, we show that in vivo knockdown of ASncmtRNAs results in drastic inhibition of tumor growth in a xenograft model of MDA-MB-231 subcutaneous tumors, further supporting this approach for the development of new therapeutic strategies against breast cancer.

## Introduction

Breast cancer (BrCa) is the leading cause of cancer mortality in women worldwide with a prevalent malignancy of 2,088,000 cases and about 626,000 deaths in 2018^1,2^. A large body of experimental evidence indicates that BrCa is a well-known case of hereditary malignancy and DNA methylation is an epigenetic modification playing an important role in BrCa development^3,4^. In addition, BrCa is a heterogeneous disease comprising three subtypes; luminal BrCa is characterized by high expression of the estrogen (ER) and progesterone receptors (PR) but not human epidermal growth factor receptor 2 (HER2); or by overexpression of HER2 and low expression of ER and PR, and high expression of HER2. A third subtype of BrCa is termed triple-negative because these tumors express neither ER, PR nor HER2^5–8^. In consequence, the response of BrCa to chemotherapeutic agents and immunotherapy is generally poor and is complicated by this heterogeneity. Thus, there is an urgent need for the development of new therapeutic strategies against BrCa^8,9^.

Human and mouse cells express a family of mitochondrial long noncoding RNAs (ncmtRNAs), comprised of sense (SncmtRNA), and antisense (ASncmtRNAs) members, which contain inverted repeats (IR) and therefore stem-loop structures^10–15^. SncmtRNA is expressed in all proliferating but not in resting cells, suggesting a role for this transcript in cell proliferation^10–12^. Normal proliferating cells also express two antisense transcripts, ASncmtRNA-1 and ASncmtRNA-2^11,12^. Remarkably, however, the ASncmtRNAs are downregulated in human and mouse tumor cell lines^11–15^. Thus, it seems that, at least in these two mammalian species, downregulation of the ASncmtRNAs is an important step in carcinogenesis and represents a new generalized pro-tumorigenic hallmark of cancer^16^.

ASncmtRNA knockdown (ASK for short) using chemically-modified antisense oligonucleotides (ASO) induces apoptotic death of a wide array of human cancer cell lines from several tissue origins^14^. In addition, ASK also induces apoptotic death of several murine tumor cells, including the highly aggressive B16F10 melanoma^12^ and the renal carcinoma RenCa cell line^13^. Moreover, in syngeneic subcutaneous B16F10 and orthotopic RenCa models, ASK drastically inhibits tumor growth and metastasis^12,13^. Of note, the same results were obtained in a lentiviral-based shRNA approach in the B16F10 syngeneic murine model^17^, providing further proof-of-concept of the ASncmtRNAs as therapeutic targets in this cancer model. Interestingly, ASK does not affect the viability of human or murine normal cells^12–14^.

At the molecular level, ASK induces downregulation of survivin (BIRC5) in several human and mouse tumor cells^12–14^. Survivin, an essential inhibitor of apoptosis (IAP) protein, is upregulated in virtually all human tumors^18^, which has inspired multiple efforts in targeting this protein for cancer therapy, with modest results^19^. Besides its function in apoptosis, survivin also plays a fundamental role at the mitotic spindle assembly checkpoint^20^ and cytokinesis^21^, thus allowing progression through the M-phase of the cell cycle. In agreement with survivin reduction upon ASK, apoptotic death of tumor cells is preceded by a drastic inhibition of cell proliferation^12–15^, suggesting that this treatment induces alteration of factor(s) involved in cell cycle progression.

In this work, we show that ASK using an ASO directed to both ASncmtRNAs, Andes-1537, induces massive death of three human breast cancer cell lines, MDA-MB-231, MCF7, and ZR-75-1. An early proliferative blockage is also observed in all three cell lines. The strong inhibition of proliferation is explained by a reduction in the levels of the key cell cycle control proteins cyclin B1, cyclin D1, CDK1, CDK4^22^, as well as survivin. Moreover, the treatment induces upregulation of miRNAs hsa-miR-1973, hsa-miR-4485-3p, and hsa-miR-4485-5p, which conserve 100% sequence identity to segments of the IR of ASncmtRNA-2^14,23^. Transfection of MDA-MB-231 cells with a hsa-miR-4485-3p mimic induces downregulation of cyclin B1 and cyclin D1, without affecting the levels of CDK1, CDK4, or survivin. In addition, small RNAseq analysis revealed upregulation of several nuclear-encoded miRNAs. Mimics of two of these, hsa-miR-5096 and hsa-miR-3609, induce reduction of CDK1. These results suggest that at least ASncmtRNA-2 plays an important function in the regulation of cell cycle progression. In addition to the effect on viability and proliferation, ASncmtRNA knockdown also induces inhibition of stemness and a drastic reduction in the invasion capacity of the three breast cancer cell lines. Translation of these results to xenografts of MDA-MB-231 cells showed a drastic delay in tumor growth in mice treated with Andes-1537, corroborating our in vitro results and further supporting a new therapeutic approach for this worldwide leading cancer in women^1,2^.

## Results

### ASK induces proliferative arrest and apoptosis of breast cancer cell lines

**Fig. 1:**
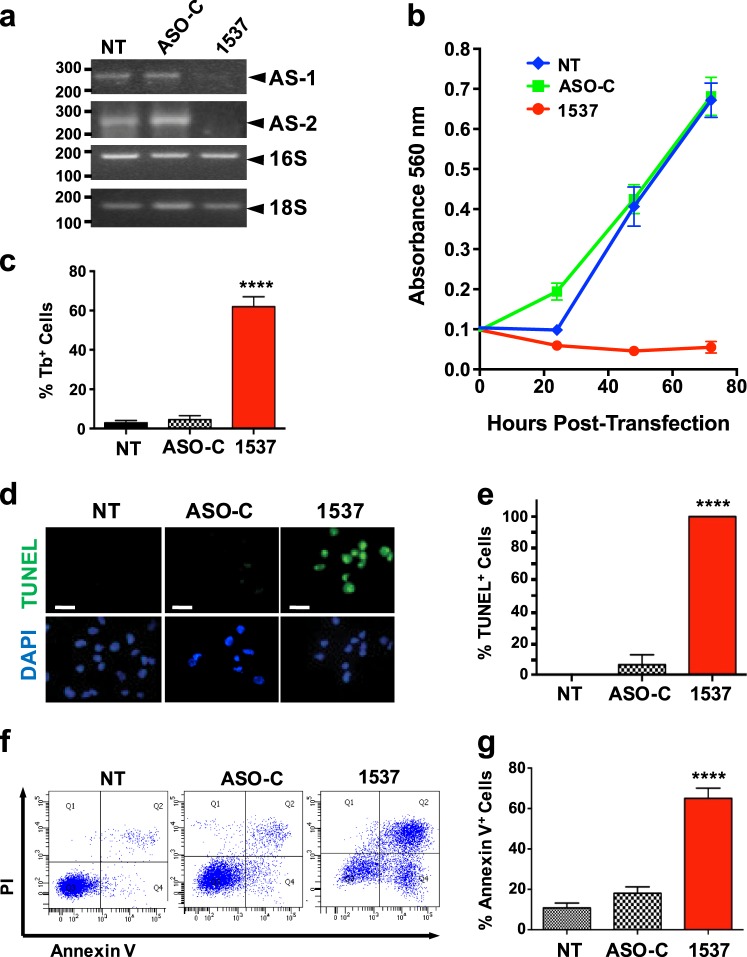
ASncmtRNA knockdown (ASK) induces inhibition of cell proliferation and apoptotic cell death of MDA-MB-231 breast cancer cells. MDA-MB-231 cells were transfected with 200 nM Andes-1537 (1537) or ASO-C or left untreated (NT). **a** Knockdown of ASncmtRNA-1 and −2 at 24 h post-transfection was corroborated by RT-PCR, using 16 S and 18 S rRNA as controls. Numbers on the left represent size in bp of MW standards. **b** Viability was determined by MTT assay at 24, 48, and 72 h post-transfection. **c** At 48 h post-transfection, Andes-1537 induced over 60% death, determined by Trypan blue (Tb) exclusion assay, compared to 3–5% in controls (two-tailed Student’s *t*-test; Mean ± S.E.M. ****p < 0.0001; ASO-C vs. Andes-1537; n = 3). **d** Representative image of fluorescent TUNEL assay for determination of apoptosis, at 48 h post-transfection. Bars = 50 µm. **e** Ten fields per sample of the experiment shown in **d** were photographed and quantified. The graph shows % of TUNEL-positive cells (two-tailed Student’s *t*-test; Mean ± S.E.M. ****p < 0.0001; ASO-C vs. Andes-1537; n = 3). **f** Apoptosis was further confirmed at 48 h post-transfection by Annexin V-binding and co-staining with PI and analyzed by flow cytometry. **g** A triplicate analysis of the experiment in **f** shows increased Annexin V binding in Andes-1537-treated cells, compared to controls (two-tailed Student’s *t*-test; Mean ± S.E.M.****p < 0.0001; ASO-C vs. Andes-1537; *n* = 3)

Transfection of MDA-MB-231 cells with Andes-1537, targeted to the human ASncmtRNAs, induced knockdown of both ASncmtRNA-1 and ASncmtRNA-2, compared to non-treated control (NT) and control ASO (ASO-C)-transfected cells (Fig. 1a). As observed previously for several human and mouse tumor cells lines^12–15^, knockdown of ASncmtRNAs induces a drastic reduction in cell viability in comparison with both controls, at least up to 72 h post-transfection, evidenced by MTT (Fig. 1b). The extent of cell death induced by Andes-1537 was significantly higher than controls, as observed by Trypan blue (Tb) exclusion assay in MDA-MB-231 at 48 h post-transfection (Fig. 1c) and this cell death occurs through apoptosis, according to TUNEL assay (Fig. 1d, e) and Annexin V-propidium iodide (PI) double-staining (Fig. 1f, g).

In order to probe the generality of this treatment in breast cancer, we performed the same determinations on two additional cell lines representing different subtypes of breast cancer, MCF7 and ZR-75-1. Western blot analysis confirmed MDA-MB-231, MCF7, and ZR-75-1 cells as triple-negative, ER-positive and HER2-positive cell lines, respectively (Supplementary Fig. 1). MTT assay showed that both MCF7 (Supplementary Fig. 2a) and ZR-75-1 (Supplementary Fig. 2b) display a drastic loss in viability up to 72 h, as observed for MDA-MB-231 cells (Fig. 1b). Similarly, both cell lines exhibited a significantly higher death rate at 48 h post-transfection (Supplementary Fig. 2c, d). Adding to its potential for cancer therapy, treatment with Andes-1537 also induced inhibition of stemness, evidenced by mammosphere formation assay (Supplementary Fig. 3) and invasive capacity of all three breast cancer cell lines (Supplementary Fig. 4). These results show the effectiveness of the treatment on all three breast cancer cell lines, regardless of the subtype.

### ASK induces proliferative arrest through downregulation of key cell cycle progression factors

**Fig. 2:**
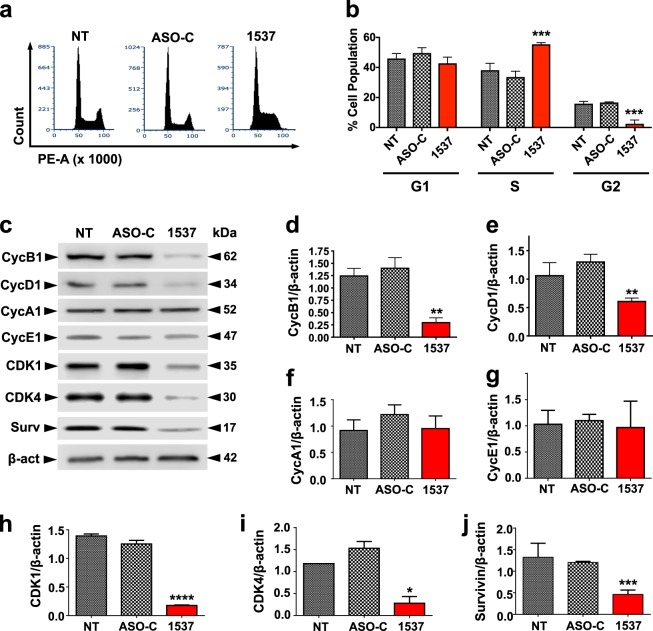
ASK induces cell cycle arrest through downregulation of key cell cycle progression factors. **a** MDA-MB-231 cells were transfected with 200 nM Andes-1537, ASO-C or left untreated (NT) for 24 h, stained with PI and cell cycle distribution was analyzed by flow cytometry. **b** A triplicate analysis of the experiment in **a** shows accumulation of cells in S phase and a decrease in the G2/M population, compared to controls (ASO-C vs. Andes-1537; two-tailed Student’s *t*-test, ***p < 0.001). **c** Representative results of Western blot analysis of cells treated as in **a** for 24 h. Surv, survivin; β-act, β-actin. Quantification of three independent experiments (*n* = 3) is shown for cyclin B1 (**d**), cyclin D1 (**e**), cyclin A1 (**f**), cyclin E1 (**g**), CDK1 (**h**), CDK4 (**i**) and survivin (**j**). **d**–**j** two-tailed Student’s *t*-test; (*p < 0.05; **p < 0.01; ***p < 0.001; ****p < 0.0001; ASO-C vs. Andes-1537). All graphs represent Mean ± S.E.M.

The time-dependent loss in viability of Andes-1537-transfected cells (Fig. 1a and Supplementary Fig. 2a, b) could be attributed not solely to an increase in death rate, but also to a decrease in proliferative index. Indeed, flow cytometric cell cycle analysis at 24 h post-transfection showed a higher accumulation of Andes-1537-treated cells in S-phase, compared to controls, with a concomitant decrease in the G2/M population (Fig. 2a, b), indicative of cell cycle arrest. At the molecular level, the observed cell cycle arrest is reflected in a strong and specific downregulation of two essential cyclins at 24 h post-transfection, cyclin B1 (Fig. 2c, d) and cyclin D1 (Fig. 2c, e), while neither cyclin A1 (Fig. 2c, f) nor cyclin E1 (Fig. 2c, g) were affected. In addition, the cyclin-dependent kinases CDK1 (Fig. 2c, h) and CDK4 (Fig. 2c, i) were also significantly reduced. Moreover, as previously shown for other human and mouse cell lines^12–14^, the treatment also induced downregulation of survivin (Fig. 2c, j), a factor which is central not only to apoptosis, but also plays a crucial role in M-phase progression^18–21^.

### miRNAs contained in the IR of ASncmtRNA-2 are upregulated upon ASK

**Fig. 3:**
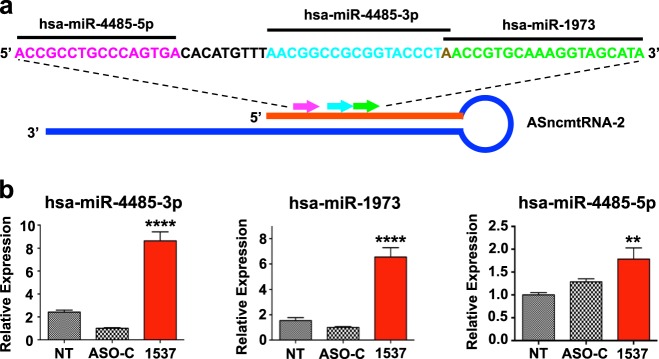
The miRNAs hsa-miR-4485-5p, hsa-miR-4485-3p and hsa-miR-1973 are upregulated by ASK. **a** Scheme depicting sequence and position of hsa-miR-4485-5p (pink), hsa-miR-4485-3p (teal), and hsa-miR-1973 (green) on the IR of ASncmtRNA-2. The A in brown represents a base that is contained in hsa-miR-4485-3p as well as in hsa-miR-1973. **b** Relative quantification of miRNAs by RT-qPCR (Taqman assay) is shown for hsa-miR-4485-5p, hsa-miR-4485-3p and hsa-miR-1973, in a triplicate analysis of three independent experiments (*n* = 3), in untreated cells (NT) or cells transfected with Andes-1537 or ASO-C at 24 h post-transfection (two-tailed Student’s *t*-test; Mean ± S.E.M. **p < 0.01; ****p < 0.0001; ASO-C vs. Andes-1537)

We previously postulated that the molecular effects of ASK on tumor cells could be mediated by miRNAs^14^. Indeed, the sequences of three known miRNAs, hsa-miR-4485-5p, hsa-miR-4485-3p, and hsa-miR-1973 (which is expressed as −3p), are completely and identically contained in the IR of ASncmtRNA-2^23^ (Fig. 3a), whereas the ASncmtRNA-1 IR does not contain any known miRNAs. As evidenced by TaqMan quantitative assays, 24 h post-transfection ASK induced an increase of 6–7 times (fold-change; FC) in the levels of hsa-miR-4485-3p and hsa-miR-1973, compared to ASO-C treatment; however, hsa-4485-5p only increased by around 30% (Fig. 3b). This result, along with the data found on miRBase (www.mirbase.org), where hsa-miR-4485-5p is much less represented than its −3p counterpart, led us to hypothesize that this mature sequence corresponds to the passenger strand of the hsa-miR-4485 duplex and was not further analyzed.

### hsa-miR-4485-3p mimic induces downregulation of cyclins B1 and D1

**Fig. 4:**
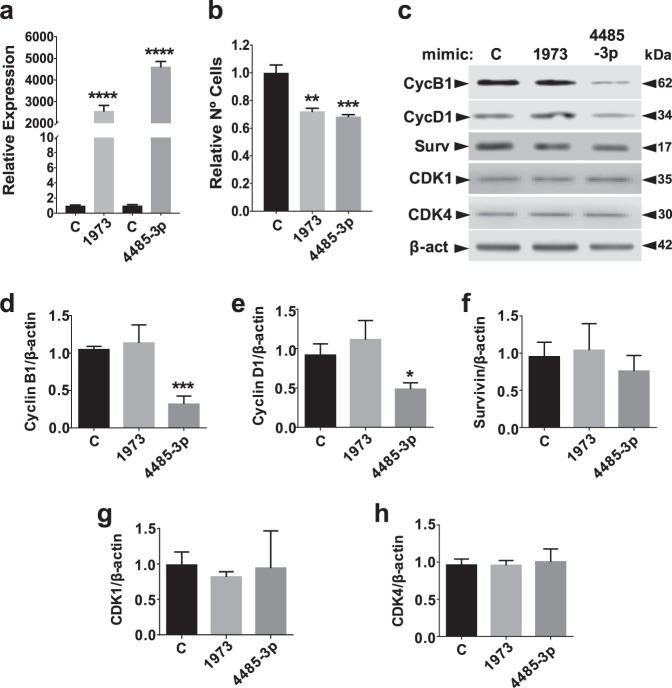
Transfection of MDA-MB-231 cells with hsa-miR-4485-3p mimic induces downregulation of cyclin B1 and cyclin D1. MDA-MB-231 cells were transfected for 48 h with control mimic (C), hsa-miR-1973 mimic (1973) or hsa-miR-4485-3p mimic (4485-3p) (*n* = 3). **a** miRNA upregulation was confirmed by Taqman RT-qPCR assays (two-tailed Student’s *t*-test; ****p < 0.0001; C vs. each specific mimic) (*n* = 3). **b** Relative total number of cells (two-tailed Student’s *t*-test; **p < 0.01, ***p < 0.001; C vs. each specific mimic; *n* = 3). **c** Representative results of Western blot analysis in mimic-transfected cells. Surv, survivin; β-act, β-actin. **d**–**h**, Graphical representation of the results of three independent experiments (n = 3) for cyclin B1 (**d**), cyclin D1 (**e**), survivin (**f**), CDK1 (**g**) and CDK4 (**h**) (two-tailed Student’s *t*-test; *p < 0.01, ***p < 0.001; C vs. each specific mimic). All graphs represent Mean ± S.E.M.

We transfected MDA-MB-231 cells for 48 h with mimics of hsa-miR-4485-3p and hsa-miR-1973 or a control mimic. Overexpression of both miRNAs was corroborated by TaqMan assay (Fig. 4a). Both mimics induced inhibition of proliferation, albeit to a much lesser extent than Andes-1537 (Fig. 4b), evidencing a partial effect of either miRNA on its own. Total protein was extracted and subjected to Western blot (Fig. 4c). Hsa-mir-4485-3p mimic induced a significant reduction in cyclin B1 (Fig. 4c, d) and cyclin D1 (Fig. 4c, e) expression, but not of other proteins downregulated by Andes-1537 transfection, namely survivin (Fig. 4c, f), CDK1 (Fig. 4c, g) and CDK4 (Fig. 4c, h). On the other hand, hsa-miR-1973 mimic failed to induce downregulation of any of these proteins.

### ASK induces upregulation of nuclear-encoded miRNAs which target CDK1

**Fig. 5:**
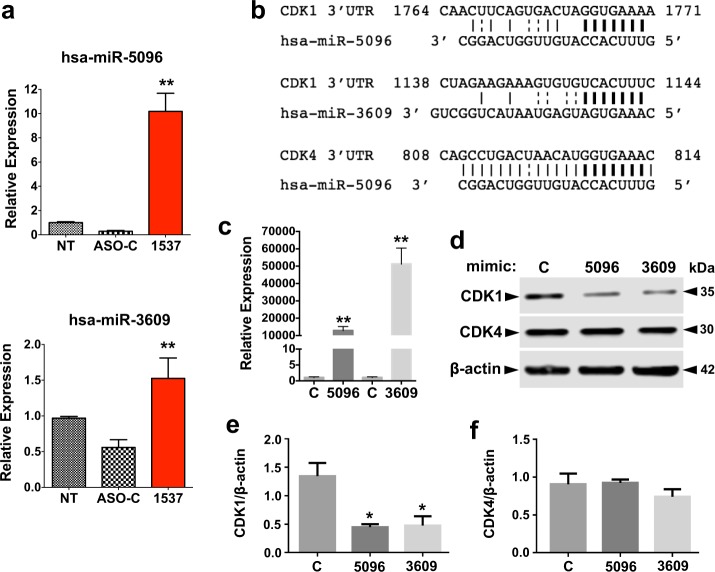
ASK induces increase of nuclear-encoded miRNAs, which regulate CDK1 expression. **a** Forty-eight hour after transfection of ASO-C or Andes-1537 or no treatment (NT), relative levels of hsa-miR-5096 and hsa-miR-3609 were determined by RT-qPCR (Taqman assays) (two-tailed Student’s *t*-test; **p < 0.01; ASO-C vs. Andes-1537; *n* = 3). **b** Putative binding sites for hsa-miR-5096 and hsa-miR-3609 on the mRNA 3’UTR of CDK1 and hsa-miR-5096 on CDK4, determined by TargetScanHuman. Bold lines represent potential 5’ seed region binding; solid lines depict Watson–Crick base pairing, broken lines show G:U wobble. **c** Relative quantification of hsa-miR-5096 and hsa-miR-3609 after transfection of mimics (two-tailed Student’s *t*-test; **p < 0.01; C vs. each specific mimic; *n* = 3). **d** Representative results of Western blot analysis for CDK1 and CDK4, using β-actin as loading control, in mimic-transfected cells at 48 h. **e**, **f** Graphical representation of triplicate analyses of the results shown in **d**, for CDK1 (**e**) and CDK4 (**f**) (two-tailed Student’s *t*-test; *p < 0.01; C vs. each specific mimic; *n* = 3). All graphs represent Mean ± S.E.M.

In order to explore additional miRNAs induced upon ASK which could be affecting cell cycle protein expression, we performed a preliminary small RNA sequencing analysis where, besides hsa-miR-4485-3p and hsa-miR-1973, we found several nuclear miRNAs that were significantly upregulated in Andes-1537-treated MDA-MB-231 cells, compared to ASO-C and untreated cells. Of these, two of the most upregulated were hsa-miR-5096 and hsa-miR-3609, which map in sequence to chromosomes 4 and 7, respectively (www.mirbase.org). Upregulation of these miRNAs was corroborated by TaqMan assays (Fig. 5a). Using TargetScanHuman, both hsa-miR-5096 and hsa-miR-3609 contain predictive canonical binding sites on the CDK1 mRNA 3’UTR, but only hsa-miR-5096 is predicted to target CDK4 (Fig. 5b). We transfected MDA-MB-231 cells with mimics of these miRNAs or control mimic, for 48 h. Overexpression of miRNAs was corroborated through TaqMan assays (Fig. 5c). Western blot showed that, indeed, both hsa-miR-3609 and hsa-miR-5095 mimics induced a significant reduction in the levels of CDK1 protein (Fig. 5d, e), but neither affected the levels of CDK4 (Fig. 5d, f), despite the extense complementarity between hsa-miR-5096 and CDK1 3’UTR (Fig. 5b). A more in-depth small RNAseq was later performed, in which we also found significant upregulation of hsa-miR-4485-3p, hsa-miR-5096 and hsa-miR-3609.

### Andes-1537 treatment in an MDA-MB-231 xenograft mouse model

**Fig. 6:**
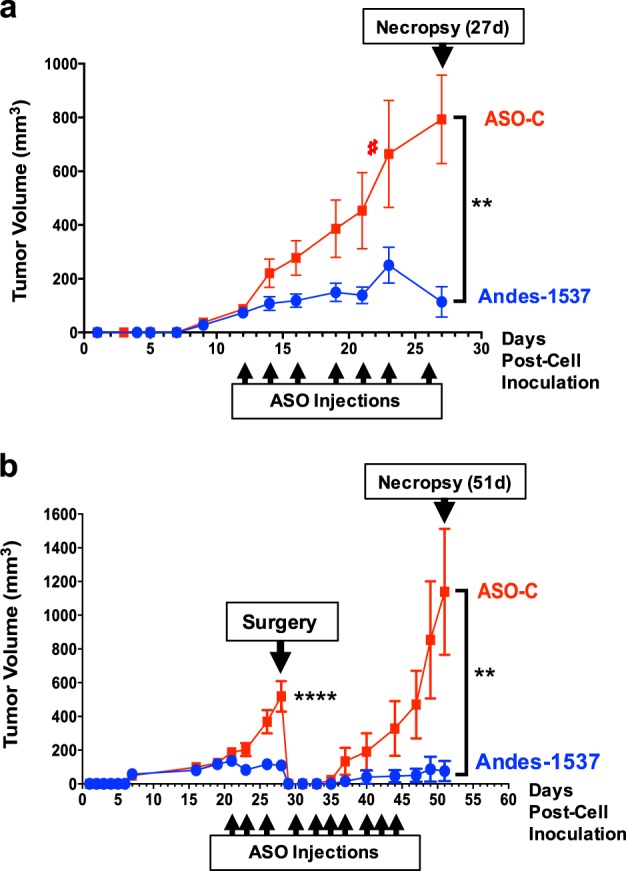
ASK retards tumor growth and precludes primary tumor relapse in an MDA-MB-231 subcutaneous xenograft assay. **a** Eleven Balb/c NOD/SCID mice were inoculated subcutaneously with 2.5 × 10^6^ cells and were then injected ip, in a blinded fashion, with ASO-C (six mice) or Andes-1537 (five mice) on days 12, 14, 16, 19, 21, 23, and 26 post-cell inoculation. Tumor size was monitored and the experiment was terminated on day 27. One mouse from the ASO-C group was sacrificed on day 23 for having reached the ethical limit for tumor size (#). **p < 0.005, two-tailed Student’s *t*-test, ASO-C vs. Andes 1537, day 27. **b** Fifteen mice were inoculated as in **a** and injected ip with ASO-C (five mice) or Andes-1537 (10 mice) on days 21, 23, 26, 30, 33, 35, 37, 40, 42, and 44 post-cell inoculation. Primary tumors were surgically resected on day 29. The experiment was terminated on day 51. **p < 0.005, ****p < 0.0001, two-tailed Student’s *t*-test, ASO-C vs. Andes-1537, day 51. Graphs represent Mean ± S.E.M.

In order to validate the use of Andes-1537 as a therapeutic tool against breast cancer, we studied the effectiveness of in vivo ASncmtRNA knockdown on tumor cell proliferation, in a xenograft assay in mice. Of the three cell lines used in this study, only MDA-MB-231 cells produced tumors in a reasonable time period (Supplementary Fig. 5). Therefore, we induced subcutaneous tumor formation in immunocompromised (NOD/SCID) Balb/c mice by inoculation of MDA-MB-231 cells. After 7 intraperitoneal (ip) injections of Andes-1537, tumor growth was drastically diminished, compared to ASO-C (Fig. 6a). We also performed a surgical assay, in which the primary tumor was resected 29 days after cell inoculation, with administration of ASOs before and after resection, simulating a therapeutic approach applied on some patients. In this assay, tumor growth was greatly reduced with Andes-1537 before, as well as after tumor resection (Fig. 6b). These results strongly suggest that ASncmtRNA knockdown inhibits tumor cell proliferation in vivo, probably mediated in part by the miRNAs described in this work.

## Discussion

As observed for several other human and mouse tumor cell lines^12–14,17^, ASK induces massive cell death in the MDA-MB-231, MCF-7, and ZR-75-1 human breast cancer cell lines (Fig. 1c, Supplementary Fig. 2c, d), despite belonging to different breast cancer subtypes. Cell death is preceded by an abrupt inhibition of proliferation^12–15^ (Fig. 1b, Supplementary Fig. 2a, b). Our data strongly suggest that these effects are brought about by miRNAs that are induced upon ASK^14^. The miRNAs whose sequences are contained in the IR of ASncmtRNA-2 are annotated on chromosome 4 (hsa-miR-1973) and 11 (hsa-miR-4485-5p and hsa-miR-4485-3p) (www.mirbase.org), respectively, but strong evidence points to their mitochondrial origin. First, the reads from our small RNAseq analyses, in addition to those on miRbase, indicate a higher proportion of mitochondrial-encoded than nuclear-encoded sequence in bases flanking these miRNAs (Supplementary Fig. 6). Furthermore, Bianchessi et al.^23^ showed that ASncmtRNA-2, along with hsa-miR-4485 and hsa-miR-1973 are drastically decreased upon treatment of cells with ethidium bromide, which affects mitochondrial DNA replication and transcription^24^, in agreement with our previous report that rhodamine 6 G, another drug that blocks mitochondrial transcription^25^, also inhibits expression of the ASncmtRNAs^11^. In addition, we reported that the ASncmtRNAs exit mitochondria to the cytosol and the nucleus^26^, suggesting a functional role for these transcripts outside the mitochondria where they can hypothetically be processed by Dicer, thereby generating hsa-miR-4485 and hsa-miR-1973.

Out of these putative mitochondrial miRNAs, only the hsa-miR-4485-3p mimic induced downregulation of two of the key cell cycle progression factors we analyzed in this work, specifically cyclins B1 and D1, without altering the levels of survivin, CDK1 or CDK4 (Fig. 4). However, hsa-miR-4485-3p does not have predictive binding sites on the 3’UTR of the mRNAs of either of these proteins, whereas hsa-miR-1973 contains a putative site for cyclin D1, according to the TargetScanHuman platform. As most miRNA target prediction algorithms, TargetScanHuman relies on canonical 5’ seed pairing in the 3’UTR of mRNAs, but increasing evidence shows that miRNAs can also target sequences on the 5’UTR and CDS^27–30^. In addition, binding is also possible at the central region and 3’ end of many miRNAs^28,31–33^. Therefore, our results could be explained by non-canonical binding of miRNAs to their targets (Supplementary Fig. S7).

ASK also induces an increase in the nuclear-encoded miRNAs hsa-miR-5096 and hsa-miR-3609 and transfection of cells with mimics of these miRNAs downregulates CDK1. This is an essential CDK, since not only does it form part of the MPF (Maturation-promoting factor, mitosis-promoting factor, or M-Phase-promoting factor) but is reported to have the capability to single-handedly drive progression through the complete cell cycle in the absence of other CDKs^34–36^. Since this is one of the most downregulated proteins upon ASK (around 6 times FC; Fig. 2c, h), it may possibly constitute a “master trigger” for the downfall of tumor cell proliferation under these conditions.

The ASncmtRNAs, as well as SncmtRNA, belong to the family of long noncoding RNAs (lncRNAs), arbitrarily defined as transcripts longer than 200 nucleotides, which are not translated into protein^37–39^. The stem-loop structures of these transcripts are not unique since transcripts with similar structures, also containing inverted repeats, have been described in mouse oocytes^40^ and in the nematode *Caenorhabditis elegans* during fasting^41^. Members of this largely heterogeneous family of transcripts have been shown to modulate key molecular processes in animals, such as cell differentiation and proliferation^42,43^. Moreover, recent studies have shown a widespread change in lncRNA expression in cancer and experimental evidence indicates that lncRNAs play essential roles in tumorigenesis and metastasis^44^ in different types of cancer, including breast^45^. Of particular interest regarding the present work are lncRNAs that are precursors of miRNAs^46,47^, which can function as oncogenes or tumor suppressors^48^, thereby controlling cell proliferation. Those findings are similar to the results reported here.

**Fig. 7:**
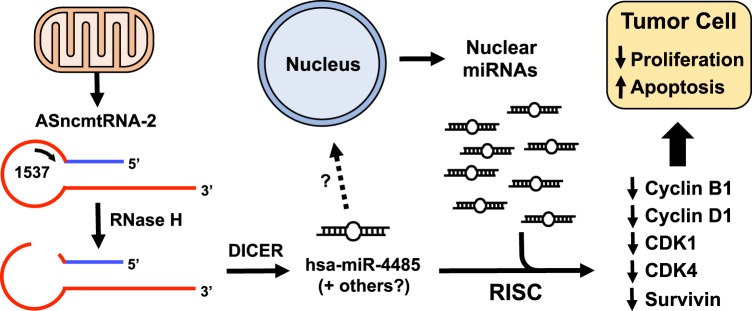
Hypothetical model for induction of proliferation blockage by ASK. ASncmtRNAs are produced in mitochondria. The antisense oligonucleotide Andes-1537 binds to the single-stranded loop region of ASncmtRNA-2, creating a substrate for RNase H, which cleaves the transcript in this region. After processing by Dicer, mitochondrial miRNA hsa-miR-4485, and possibly others, are released. By an unknown mechanism, possibly an indirect effect of hsa-miR-4485 expression, several nuclear-encoded miRNAs are increased, mainly hsa-miR-5096 and hsa-miR-3609. In conjunction, all these miRNAs block translation of key cell cycle progression factors, resulting in a drastic inhibition of proliferation. Triggering of apoptosis is mediated by miRNAs targeting survival factors such as survivin

Our results show that ASK triggers an effect that alters the levels of several key cell cycle progression proteins, mediated through the induction of mitochondrial and nuclear miRNAs, which target these proteins. A proposed model for the mechanism by which ASK causes the observed molecular effects is shown in Fig. 7. Processing of ASncmtRNA-2 by RNase H results in Dicer-mediated release of the mitochondrial miRNA hsa-miR-4485 (and perhaps others), which, in combination with nuclear miRNAs that are also induced by ASK, inhibit translation of mRNAs of key cell cycle regulators. At present, the mechanism by which ASK induces upregulation of nuclear miRNAs is unknown and further studies to this end are under way.

Taken together, the present results contribute to the understanding of the mechanisms underlying the cell cycle arrest that precedes apoptotic death of tumor cells brought about by knockdown of ASncmtRNAs and sheds light on the role of this family of transcripts in cell cycle progression. This knowledge will be essential in the light of the development of a safe and effective therapeutic strategy against breast cancer based on this approach. Indeed, we observed a strong inhibition of tumor growth in murine subcutaneous xenograft assays of MDA-MB-231 cells (Fig. 6), correlating nicely with our in vitro results and further supporting this strategy for a breast cancer therapeutic alternative. Based on these and previous results obtained with other tumor types, we recently completed a Phase Ia Clinical Trial (NCT02508441) with Andes-1537 treatment in 16 terminal patients in San Francisco, CA. Andes-1537 was well-tolerated and two patients, one with pancreatic cancer and another with cholangiocarcinoma, maintained stable disease beyond six months after beginning of treatment^49^.

## Materials and methods

### Animal studies

Animal studies were conducted in accordance with the guidelines of Comisión Nacional de Investigación Científica y Tecnológica (Conicyt), Chile, and approved by the Ethical Committee of Fundación Ciencia & Vida. Balb/c NOD/SCID mice of 6–8 weeks of age were obtained from The Jackson Laboratory, (Bar Harbor, ME) and maintained in the pathogen-free facility (Tecniplast, Buguggiate, Italy) of the Fundación Ciencia & Vida in a temperature-controlled room with a 12/12 h light/dark schedule with sterile food and water *ad libitum*.

### Cell culture and transfection

The human breast cancer cell lines MDA-MB-231 (HTB-26), MCF7 (HTB-22), and ZR-75-1 (CRL-1500) were purchased from ATCC (Manassas, VA, USA) and cultured in a humidified cell culture chamber in RPMI (Thermo Fisher Scientific, Waltham, MA, USA) supplemented with 10% fetal calf serum (Thermo Fisher Scientific) and Anti-Anti (Thermo Fisher Scientific), at 37 °C under a 5% CO_2_ atmosphere. Cultures were routinely checked for mycoplasma contamination using the EZ-PCR Mycoplasma Test kit (Biological Industries, Cromwell, CT, USA). Cultures were not used beyond 6 months after thawing. Al experiments were performed within 2 years after purchase of cell lines. For ASO transfection experiments, cells were seeded at 50,000 cells/well in a 12-well-plate (Nunc, Thermo Fisher Scientific). On the next day, cells were transfected with 200 nM (or 300 nM for ZR-75-1) ASO control (ASO-C: 5’-AGGTGGAGTGGATTGGGG) or Andes-1537 (5’-CACCCACCCAAGAACAGG) and 2 μg/ml Lipofectamine2000 (Invitrogen) or left untreated for 24 h. All ASOs contained 100% phosphorothioate internucleosidic bonds (Integrated DNA Technologies, Coralville, IA, USA). For mimic transfection, cells were seeded as above and transfected the next day with 0.5 nM control or specific mimic (Exiqon, Qiagen, Hilden, Germany) and 1 μg/ml Lipofectamine2000 (Thermo Fisher Scientific) and left for 48 h before processing.

### Cell viability

Total cell number and viability was determined by Trypan blue (Tb) or propidium iodide (PI) exclusion. PI was added at 50 μg/ml 1 min before flow cytometry on a BD Biosciences FACS Canto Flow Cytometer (Fundación Ciencia & Vida). For Tb, the number of viable and dead cells was determined counting at least 100 cells per sample in triplicate under an Olympus BX-53 fluorescence microscope. Relative cell number was determined using MTT Assay (Promega, Madison, WI, USA), according to manufacturer’s instructions.

### Apoptosis

DNA fragmentation was determined by the Dead End TM Fluorometric TUNEL kit (Promega), according to manufacturer’s directions. At least ten fields per sample were analyzed under an Olympus BX-53 fluorescence microscope. Phosphatidylserine exposure was determined by Annexin-V binding with the APOtarget kit (Thermo Fisher Scientific), according to manufacturer’s directions, co-stained with PI and analyzed by Flow cytometry on a BD Biosciences (San Jose, CA, USA) FACS Canto Flow Cytometer.

### Stemness and invasion of breast cancer cell lines

To determine stemness, Tb-negative MDA-MB-231 (2500), MCF7 (1500), and ZR-5-1 (3000) cells transfected as described above for 48 h, were suspended in MEGM medium supplemented with 25 ng/ml EGF, 5 mg/mL hydrocortisone, 5 µg/ml insulin (Lonza, Basel, Switzerland), bFGF 25 ng/ml (Thermo Fisher Scientific) and seeded into 2% agarose-coated 12-well-plates. After incubation at 37 °C for 7 days, spheres >70 μm in diameter were scored. For matrigel invasion assay, 2 × 10^5^ Tb-negative cells after a 48 h transfection were seeded over Matrigel-coated inserts (Matrigel Invasion Chamber 8.0 lm; BD Biosciences). After 24 h in culture, inserts were fixed in 4% formaldehyde and membranes were stained with DAPI, mounted in Mowiol and observed under an Olympus CKX41 microscope at ×40 magnification. At least 10 fields were evaluated.

### Cell cycle analysis

Cells transfected with Andes-1537 or ASO-C or untreated for 24 h were trypsinized, collected, fixed in 75% ethanol for 24 h, washed twice in ice-cold PBS and incubated for 30 min at 37 °C in staining solution (3.8 mM sodium citrate, 0.5 μg/ml RNase A, and 50 μg/ml PI). PI-stained cells were analyzed on a BD Biosciences FACS Canto Cytometer, using the BD FACSDIVA V8.0.1 software for acquisition and the FCS Express software for cell cycle distribution.

### Western blot

Cells transfected with ASO or mimic were trypsinized, harvested, washed in ice-cold PBS and sedimented at 1000 × *g* for 10 min at RT. Pellets were suspended in RIPA buffer (10 mM Tris-HCl, pH 7.4, 1% sodium deoxycholate, 1% Triton X-100, 0.1% SDS) containing 1 mM PMSF and protease inhibitor mixture (Sigma–Aldrich, St. Louis, MO, USA). Protein concentration was quantified using Bradford Reagent (Merck, Darmstadt, Germany). Proteins (30 μg/lane) were resolved by SDS-PAGE and transferred to polyvinylidenedifluoride membranes (Bio-Rad, Hercules, CA, USA) on a Trans-Blot Turbo Transfer System (Bio-Rad). Membranes were probed with antibodies against survivin (rabbit polyclonal 1:1000; R&D Systems, Minneapolis, MN, USA), cyclin B1 (mouse monoclonal 1:500; BD Biosciences), cyclin D1 (rabbit monoclonal 1:1000; Cell Signaling Technology, Danvers, MA, USA), cyclin A1 (rabbit polyclonal 1:1000; R&D Systems), cyclin E1 (mouse monoclonal 1:1000; Cell Signaling Technologies), CDK1 (rabbit monoclonal 1:1000; Cell Signaling Technologies), CDK4 (rabbit monoclonal 1:1000; Cell Signaling Technologies), estrogen receptor (rabbit polyclonal 1:300; Cell Signaling Technologies), Her2 (rabbit polyclonal 1:500; Cell Signaling Technologies), GAPDH (mouse monoclonal 1:2000; Abcam, Cambridge, UK), or β-actin (mouse monoclonal 1:2000; Abcam). Primary antibody binding was detected with peroxidase-labeled polyclonal anti-mouse (1:5000; Merck) or anti-rabbit IgG (1:5000; Merck) and revealed with the EZ-ECL system (Biological Industries) on a C-DiGit Blot Scanner (LI-COR Biosciences, Lincoln, NE, USA). The pixel intensity of each protein band was quantified using ImageJ software (NIH).

### Conventional and quantitative RT-PCR amplification

RNA was extracted with TRIzol reagent (Thermo Fisher Scientific) according to manufacturer’s directions. To eliminate genomic and mitochondrial DNA contamination, RNA preparations were treated with TURBO DNA-free (Thermo Fisher Scientific) according to manufacturer’s instructions. RNA integrity was assessed on an Experion Automated Electrophoresis System (Bio-Rad) and only high quality RNA was used (RQ value > 7). For conventional RT-PCR, reverse transcription was carried out with 50 ng RNA, 100 ng random hexamers, 0.5 mM each dNTP, 2 U/μl RNase-out (Thermo Fisher Scientific), 3 mM MgCl_2_, and 200 U Improm II reverse transcriptase (Promega) in a final volume of 20 μl. Reactions were incubated at 25 °C for 10 min, 42 °C for 50 min and 70 °C for 15 min. If not PCR-amplified immediately, cDNA was stored at −80 °C until use. PCR was carried out in 25 μl containing 2 μl cDNA, 0.5 mM each dNTP, 1.5 mM MgCl_2_, 2 U GoTaq (Promega), and 1 μM each forward (for) and reverse (rev) primer in the appropriate buffer. ASncmtRNA-1 and −2 were amplified using a protocol consisting of 5 min at 94 °C, 35 cycles of 94 °C, 58 °C, and 72 °C for 30 s each and a final extension at 72 °C for 10 min. Loading controls 18 S and 16 S rRNAs were amplified with the same protocol, but only for 15 and 17 cycles, respectively. The sequences of the primers used (Integrated DNA Technologies) were: 5’TAGGGATAACAGCGCAATCCTATT (forward for ASncmtRNA-1), 5’ACCGTGCAAAGGTAGCATAATCA (forward for ASncmtRNA-2 and 16 S), 5’AATAGGATTGCGCTGTTATCCCTA (reverse for 16 S), 5’CCGTAAATGATATCATCTCAACT (reverse for ASncmtRNA-1 and −2), 5’GTAACCCGTTGAACCCCATT (forward for 18 S), and 5’CATCCAATCGGTAGTAGCG (reverse for 18 S). For quantitative RT-PCR amplification of miRNAs, cDNA was synthesized with 10 ng total RNA, using the Taqman MicroRNA Reverse Transcription kit (Applied Biosystems, Foster City, CA, USA), according to manufacturer’s guidelines. PCR was carried out using Taqman Universal PCR Master Mix II in combination with TaqMan microRNA assays (Thermo Fisher Scientific), according to manufacturer’s directions, on a Stratagene Mx3000P real-time thermal cycler. Amplification was performed at 50 °C for 2 min, 95 °C for 10 min, followed by 40 cycles of 95 °C for 15 s and 60 °C for 1 min.

### Small RNAseq

Cells were transfected with ASO-C, Andes-1537 for 24 h or left untreated, after which RNA was purified as described above. Total RNA quality was assessed on an Agilent Bioanalyzer using an Agilent RNA 6000 Nano chip and only samples with a RIN value > 7 were used. Small RNA libraries were prepared using the TruSeq Stranded Small RNA Library Prep Kit (Illumina, San Diego, CA, USA), according to manufacturer’s guidelines and sequencing was performed on an Illumina MiSeq instrument, obtaining 5–10 million reads per sample. The second RNAseq was performed under the same conditions as above, in triplicate. Libraries were constructed using the NEBNext multiplex small RNA kit (New England Biolabs, Ipswich, MA, USA), following standard manufacturer’s protocols and sequencing was performed on an Illumina HiSeq2000 sequencer (20–30 million reads per sample). Reads were mapped to the human genome (GRCh38 assembly) and mapped reads were quantified using miRDeep2 (v2.0.0.8)^50^. In order to determine the most significant miRNAs that were upregulated by ASK, but also displaying significant abundance, we first determined the fold-change (FC) by dividing the mean abundance in reads per million of Andes-1537/ASO-C and filtered out miRNAs with FC < 2. We then established the percentile of each miRNA in terms of FC and abundance and multiplied both these parameters. We chose those miRNAs that displayed a score > 0.7 and compared the results from both small RNAseq experiments, resulting in 15 miRNAs (including hsa-miR-4485-3p) that fit these conditions in both. The datasets of both RNAseq experiments have been deposited in ArrayExpress (www.ebi.ac.uk/arrayexpress) under accessions E-MTAB-6762 (first RNAseq) and E-MTAB-6768 (second RNAseq).

### Search for canonical and non-canonical binding sites

miRNA target mining was performed with TargetScanHuman 7.1 target mining software (www.targetscan.org). In order to search for putative interactions based on non-canonical binding not identified with TargetScanHuman, the complete mRNAs of cyclin B1 (CCNB1; accession NM_031966.3) and cyclin D1 (CCND1; accession BC023620.2) were aligned to the reverse complement of hsa-miR-4485-3p, using Clustal Omega (https://www.ebi.ac.uk/Tools/msa/clustalo/) and the Blast algorithm (https://blast.ncbi.nlm.nih.gov/Blast.cgi), set to search for short sequences. Additionally, a target search was performed on the complete mRNAs of both genes, using the miRMap platform (www.mirmap.ezlab.org).

### Xenograft studies

To determine the antitumor effect of Andes-1537, 11 NOD/SCID female mice 6–8 weeks of age were injected subcutaneously (sc) under anesthesia with 2.5 × 10^6^ MDA-MB-231 cells on the right flank. When tumors reached a volume of about 100 mm^3^, mice were randomized into two groups of five and six animals, and received a total of 7 ip injections of 100 μl saline containing 100 μg either Andes-1537 (group of 5) or ASO-C (group of 6) on days 12, 14, 16, 19, 21, 23, and 26 post-cell inoculation, in a blinded fashion. Tumor growth was monitored with a caliper and tumor volumes were calculated following the formula: tumor volume = length × width^2^ × 0.5236. Mice were sacrificed under anesthesia on day 27 after cell injection.

In a second approach, 15 NOD/SCID female mice were injected also with 2.5 × 10^5^ MDA-MB-231 cells as described above. When tumors reached about 100 mm^3^, mice were randomized into two groups of 10 and five mice, which received 3 ip injections of 100 μl saline containing 100 μg Andes-1537 (group of 10) or ASO-C (group of 5) on days 21, 23, and 26, in a blinded fashion. On day 29, post-cell inoculation, mice were subjected to surgery under anesthesia to remove tumors, the wound was washed with 250 μl saline containing 100 μg either Andes-1537 (group of 10 mice) or ASO-C (group of five mice) and the wound was then sutured. Afterwards, mice received seven additional ip injections of either Andes-1537 or ASO-C (same groups) as described above, on days 30, 33, 35, 37, 40, 42, and 44 post-cell inoculation. Tumor growth was determined as above and all mice were sacrificed under anesthesia on day 51.

### Statistical analysis

Experiments were performed at least in triplicate. Results were analyzed by two-tailed Student’s *t*-test and represent the mean ± S.E.M. Significance (*P*-value) was set at the nominal level of *p* < 0.05 or less.

## Supplementary information

Supplementary Material
