# Supplementary material for: Mitochondrial ncRNA targeting induces cell cycle arrest and tumor growth inhibition of MDA-MB-231 breast cancer cells through reduction of key cell cycle progression factors
Source: Cell Death Dis. 2019 May 29;10(6):423. doi: 10.1038/s41419-019-1649-3 (PMC6541642; doi:10.1038/s41419-019-1649-3)
Supplement: Supplementary file 1 — Supplementary Material [file 41419_2019_1649_MOESM1_ESM.pdf]

**Supplementary Figures**

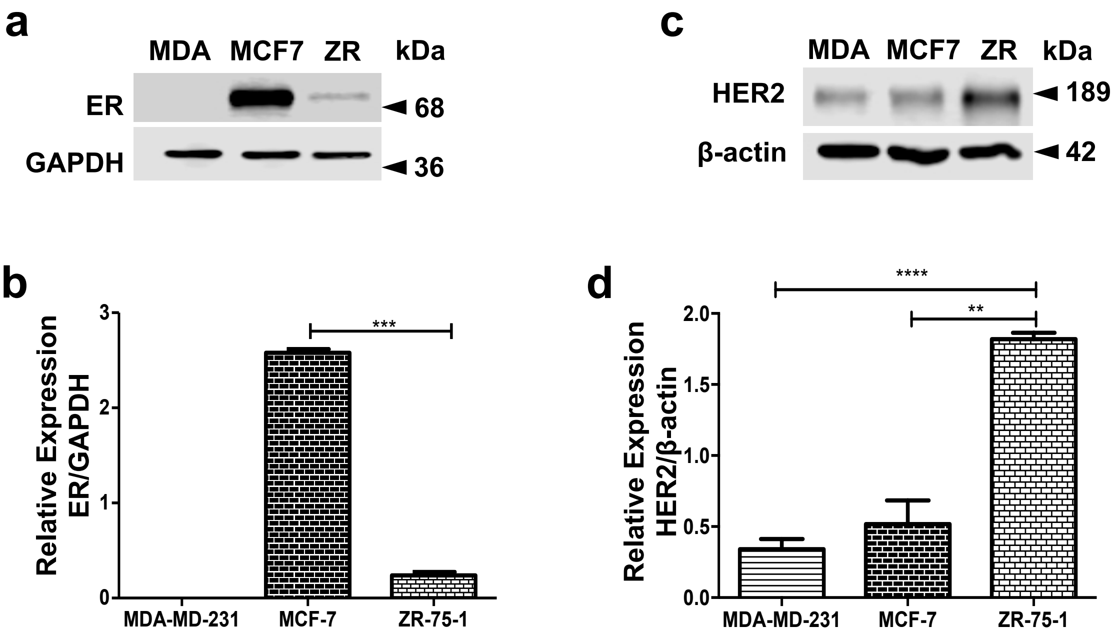


**Supplementary Figure 1. Characterization of breast cancer cell lines.** Lysates of MDA-MB-231 (MDA), MCF7 and ZR-75-1 (ZR) cells were analyzed by Western blot with antibodies against ER and HER-2. **a** Representative Western blot image for detection of ER, using GAPDH as loading control. **b** A triplicate analysis of the experiment in **a** showed overexpression of ER only in MCF-7 cells (***$p$<0.0001). **c** Representative Western blot image for detection of HER2, using β-actin as loading control. **d** A triplicate analysis of the experiment in **c** showed that HER-2 was overexpressed in ZR-75-1 cells (**$p$=0.005; ****$p$<0.0001).

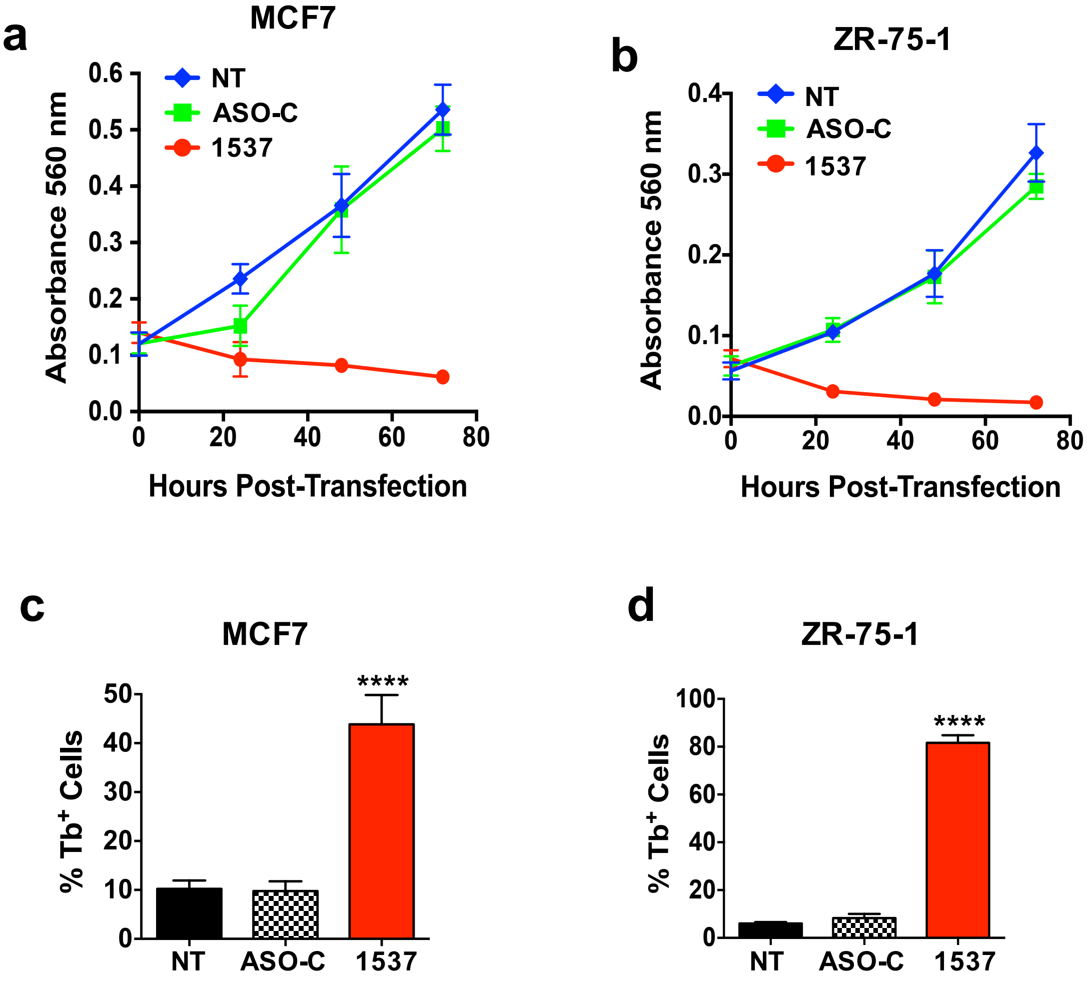


**Supplementary Figure 2. ASK induces inhibition of cell proliferation and death of two other breast cancer cell lines.** MCF7 and ZR-75-1 cells were transfected with Andes-1537 (1537) or ASO-C or left untreated (NT). Viability was determined by MTT assay at 24, 48 and 72 h post-transfection, showing a drastic decrease for both MCF7 (**a**) and ZR-75-1 (**b**). At 24 h post-transfection, cell death was determined by Trypan blue (Tb) exclusion assay. **c** MCF7 cells exhibited around 45% death by Andes-1537 treatment, compared to 10% in controls (two-tailed Student's *t*-test; Mean ± S.E.M. ****$p$<0.0001; ASO-C vs. Andes-1537; n=3). **d** Andes-1537 induced over 80% death in ZR-75-1 cells, compared to 5-8% in controls (two-tailed Student's *t*-test; Mean ± S.E.M. ****$p$<0.0001; ASO-C vs. Andes-1537; n=3).

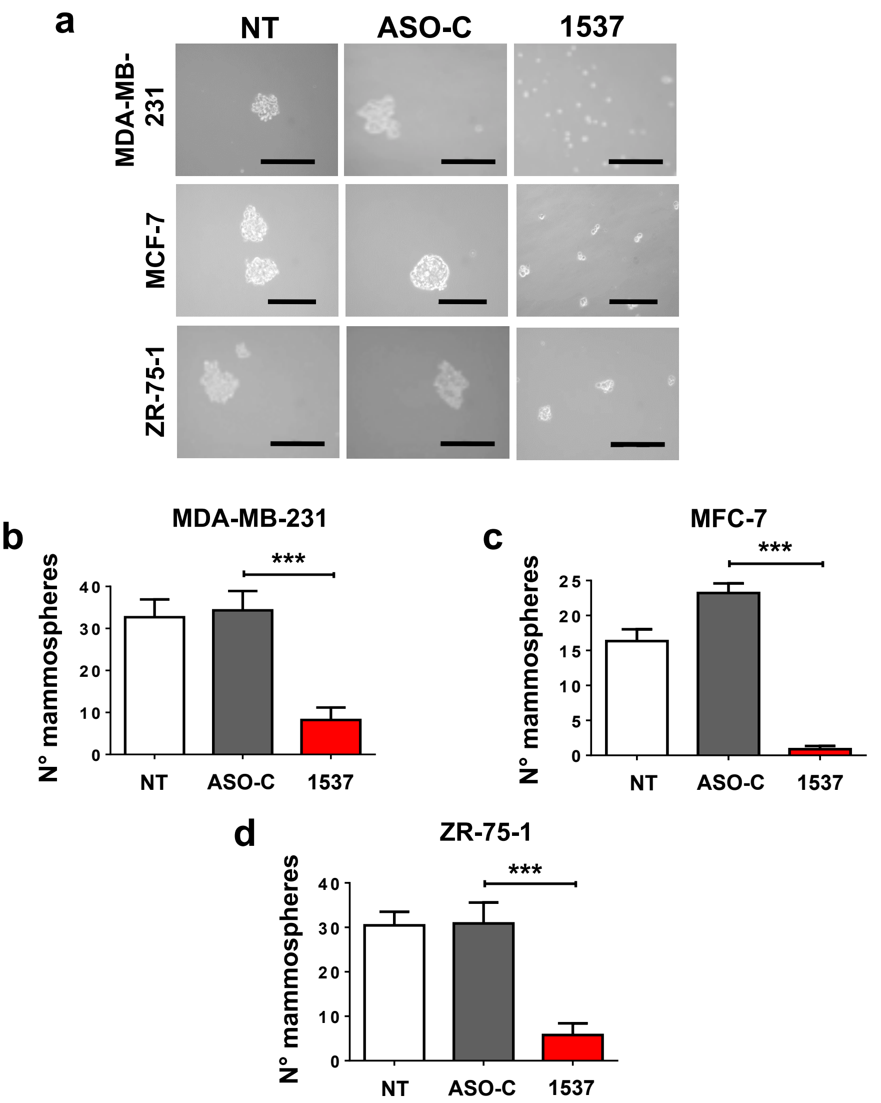


**Supplementary Figure 3. ASK precludes mammosphere formation.** MDA-MB-231, MCF7 and ZR-75-1 cells (50 000 each) were seeded into 12-well plates for 12 h and transfected with Andes-1537 or ASO-C or left untreated (NT) for 48 h. After harvesting, 5,000 Tb−negative cells were seeded into 12-well agarose-coated plates and cultured for 10-12 days, when spheres >70 µm in diameter were scored. **a** Representative phase contrast images of spheres (Bars = 200 µm). **b-d** Triplicate analysis showing the inhibitory effect of ASK on mammosphere formation for MDA-MB-231 (b, ***$p$=0.0002), MCF7 (c, ***$p$<0.0001) and ZR-75-1 (d, ***$p$=0.0002) cells.

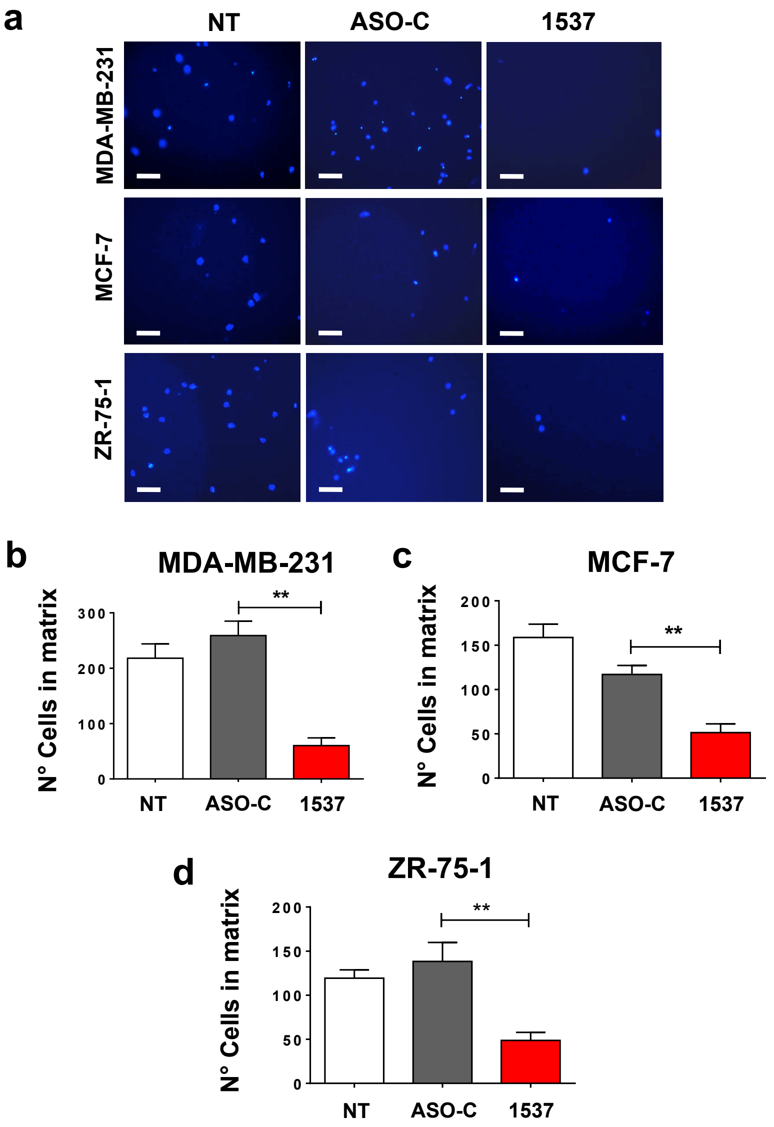


**Supplementary Figure 4. ASK reduces invasiveness of breast cancer cell lines. a** Matrigel invasion assay of MDA-MB-231, MCF7 and ZR-75-1 cells. Cells were transfected with Andes-1537, or ASO-C or left untreated (NT) for 24 h and invasion was determined with Matrigel invasion assay. Bars = 100 µm. **b-d** A triplicate analysis showed that Andes-1537 induces a drastic inhibition of the invasive capacity of MDA-MB-231 (**b**, ***p*=0.0058), MCF7 (**c**, ***p*=0.004) and ZR-75-1 (**d**, ***p*=0.022) cells.

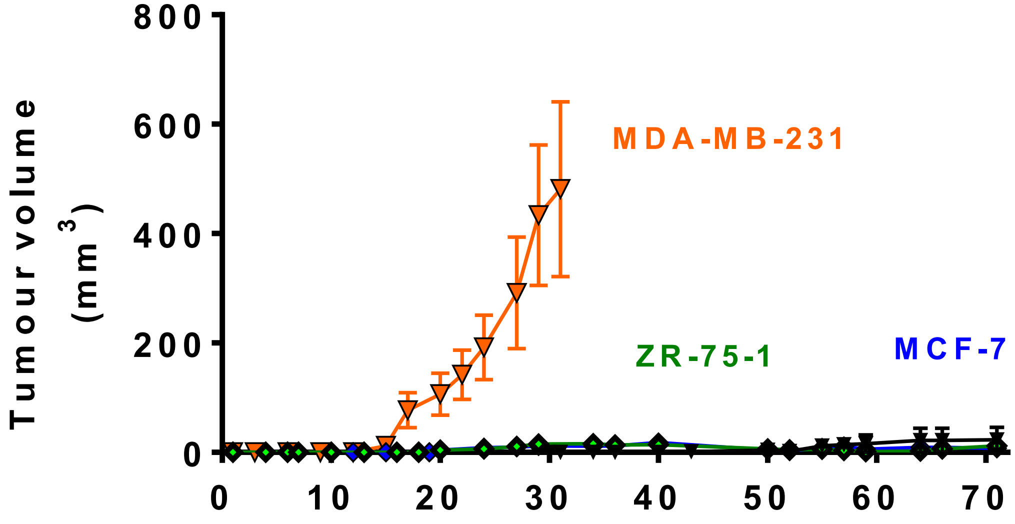


**Supplementary Figure 5. Titration of breast cancer cell lines in a xenograft murine model.** 2,5 x $10^6$ MDA-MB-231, MCF7 or ZR-75-1 cells were injected sc in the left flank of NOD/SCID mice and tumor growth was monitored with a caliper. Compared with the other two cell lines, MDA-MB-231 cells generate a more aggressive tumor growth.

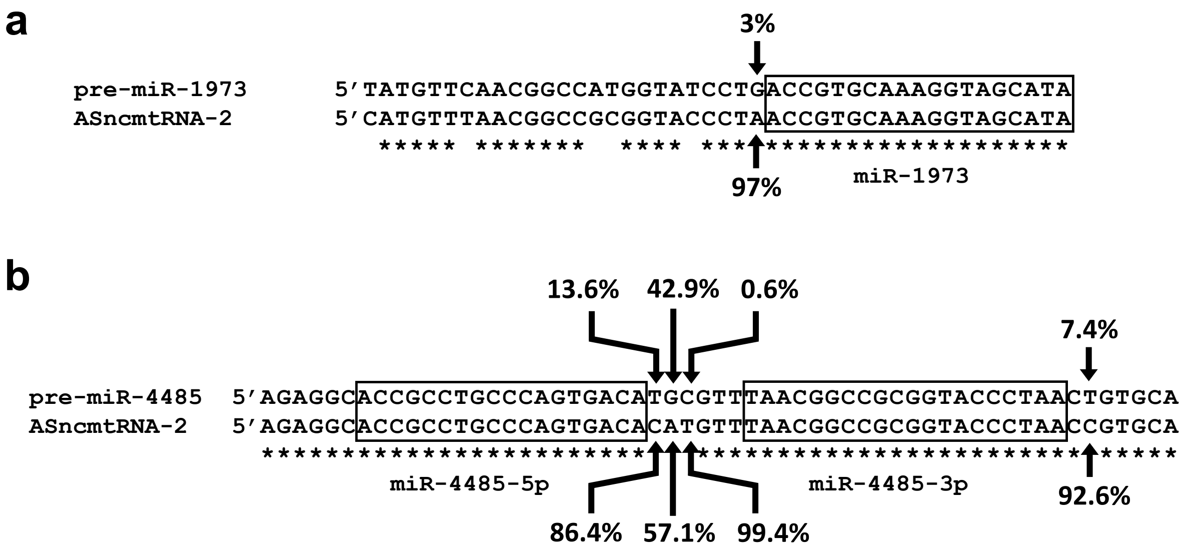


**Supplementary Figure 6. Sequences flanking mature miRNAs hsa-miR-1973, hsa-miR-4485-5p and hsa-miR-4485-3p indicate their mitochondrial origin.** Sequences of pre-miR-1973 (**a**) and pre-miR-4485 (**b**) are shown, aligned to the homologous regions in the IR of ASncmtRNA-2. Although the sequences of the mature miRNAs (boxed) are identical, the flanking regions in the putative pre-miRNAs contain several differences. For each case, the frequency of each base obtained from our RNA-seq reads and public data is shown for the putative nuclear-encoded pre-miRNA and the homologous position in ASncmtRNA-2 IR. Sequence identity is marked by asterisks.

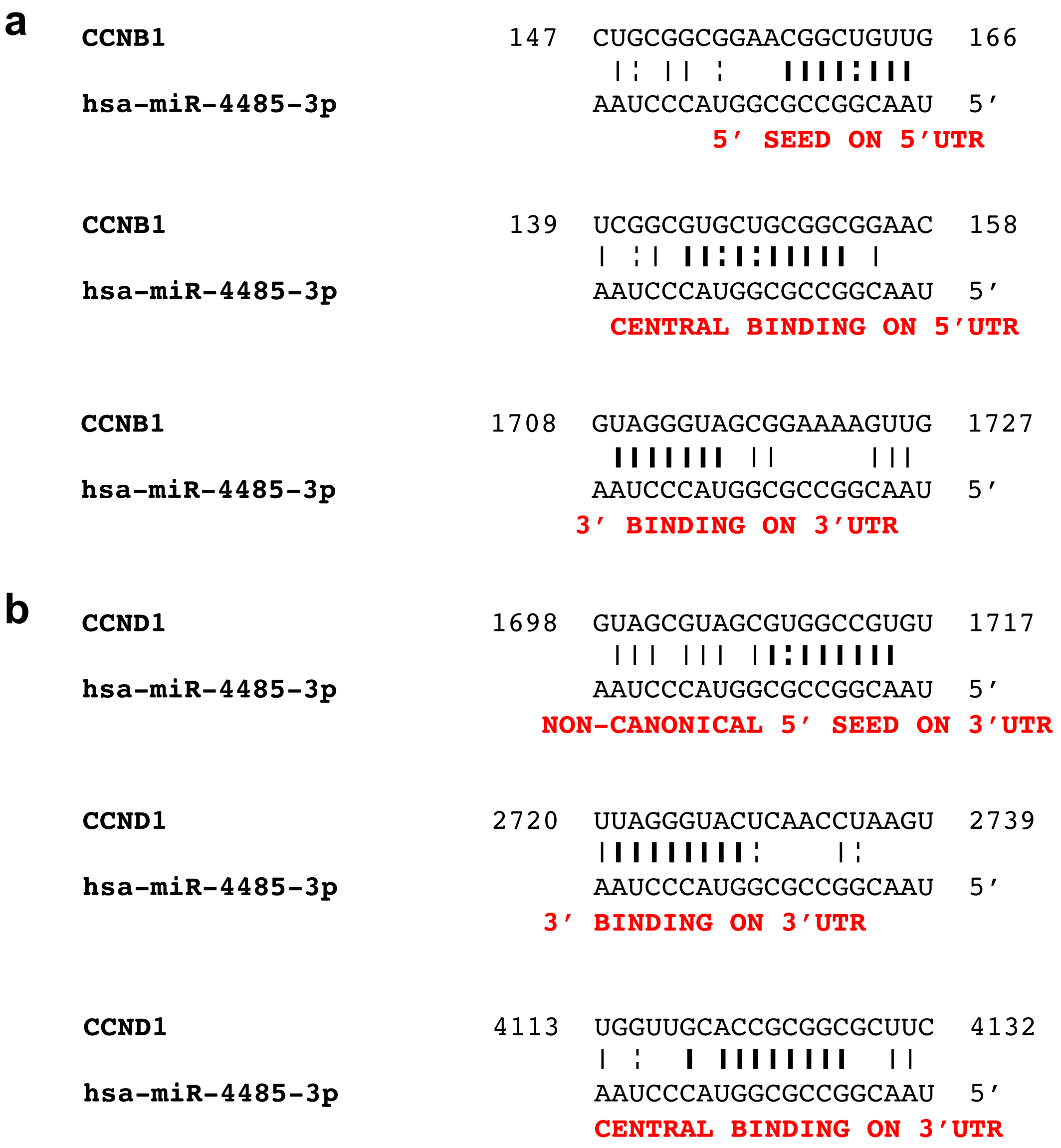


**Supplementary Figure 7. Putative non-canonical binding sites for hsa-miR-4485-3p on the mRNA of cyclin B1 and cyclin D1. a** Putative non-canonical binding sites are shown for cyclin B1 mRNA, as predicted with miRMap (first alignment) and Blast (second and third alignment). **b** Putative sites on cyclin D1 mRNA determined by Blast alignment. Bold lines denote predicted “seeds”; solid lines represent Watson-Crick pairing and broken lines are G:U wobble pairs.
